# Human leukocyte antigen association in systemic sclerosis patients: our experience at a tertiary care center in North India

**DOI:** 10.3389/fimmu.2023.1179514

**Published:** 2023-09-13

**Authors:** Sanghamitra Machhua, Shefali Khanna Sharma, Yashwant Kumar, Surjit Singh, Ritu Aggarwal, Shashi Anand, Manoj Kumar, Heera Singh, Ranjana Walker Minz

**Affiliations:** ^1^ Department of Immunopathology, Post Graduate Institute of Medical Education and Research, Chandigarh, India; ^2^ Department of Internal Medicine, Postgraduate Institute of Medical Education and Research, Chandigarh, India; ^3^ Allergy Immunology Unit, Department of Pediatrics, Advanced Pediatrics Centre, Post Graduate Institute of Medical Education and Research, Chandigarh, India

**Keywords:** SSc, HLA, lcSSc, dcSSc, phenotype

## Abstract

**Introduction:**

Systemic sclerosis (SSc) is a chronic multisystem autoimmune rheumatic disease of unknown etiology. Several studies have established that SSc is triggered by a dynamic interplay between genetic factors and environmental stimuli. In the present study, we aimed to study the association of human leukocyte antigen (HLA) with familial and non-familial SSc patients [limited cutaneous SSc (lcSSc) and diffuse cutaneous SSc (dcSSc)] from North India.

**Methods:**

The HLA-A, B, DRB1, and DQB1 genotyping of 150 (70 lcSSc and 80 dcSSc) adult-onset SSc patients and 150 age-gender-matched healthy controls were performed with sequence-specific oligonucleotide (SSO) typing kits using the luminex platform. HLA typing for HLA class I (A, B, and C) and II (DRB1, DQB1, and DPB1) in five North Indian families consisting of parent–child/sibling pairs affected with SSc or overlap syndrome was performed by Next Generation Sequencing (NGS) with Illumina MiniSeq.

**Rseults:**

Among the non-familial SSc patients, HLA- DRB1*11 (*P* = 0.001, OR: 2.38, *P*_c_ = 0.01) was identified as a risk allele, and DRB1*12 (*P* = .0001, OR: 0.00, *P*_c_ = 0.001) as a protective allele. There was no statistical association found with HLA-DQB1*. Also, no significant association was observed between HLA antigens and different clinical subsets (lcSSc and dcSSc) of SSc. Two cases of familial SSc patients had the DRB1*11 allele. The DRB1*12 allele was absent in all the familial SSc patients.

**Discussion:**

HLA DRB1*11 (risk allele) and DRB1*12 (protective allele) were found to be strongly associated with non-familial SSc patients and partially explain the disease’s familial clustering, supporting the susceptible genetic background theory for SSc development. The study also indicates the HLA allele as a common genetic risk factor in distinct autoimmune diseases contributing to overlap syndrome or polyautoimmunity.

## 1 Introduction

Systemic sclerosis (SSc) is a rare autoimmune rheumatic disease of unknown etiology characterized by excessive extracellular matrix deposition leading to fibrosis of the skin and visceral organs (1). Patients with SSc are classified into two subsets, limited cutaneous systemic sclerosis (lcSSc) and diffuse cutaneous systemic sclerosis (dcSSc), based on the extent of their skin involvement. SSc is heterogeneous in its clinical presentation that likely reflects different genetic or triggering factors (i.e., infectious agents such as cytomegalovirus and Epstein-Barr virus or environmental toxins such as organic solvents, toluene, xylene, trichloroethylene, and polyvinyl chloride) (2), which results in a chronic and self-amplifying process marked by vascular changes, inflammation, autoimmunity, and fibrosis. Endothelial cells, platelets, structural cells, immune cells, and other cell types are all involved in the disease process. These cells are activated by mediators such as transforming growth factor-β, interleukins, endothelin 1, serum autoantibodies, and reactive oxygen species (1, 2). The pathogenesis of SSc is still poorly understood. As per the literature, SSc develops in an individual with a “permissive” genetic background (2). Although SSc is not inherited in a Mendelian fashion, the relative risk of developing SSc is much higher (1.6%) in families with a history of SSc *versus* the general population (0.026%) (3). The familial history of SSc increases the risk of developing SSc in siblings (13- to 15-fold) and first-degree relatives (15- to 19-fold) compared with the general population (3). Hudson M et al. reported that 38% of SSc patients had at least one or other overlapping autoimmune disease (AID), suggesting AIDs share genetic risk factors and the role of genetic influences in SSc (4). The major histocompatibility complex (MHC) or human leukocyte antigen (HLA), located on chromosome 6p21.3, represents the densest and polymorphic region of the human genome and is observed to have the strongest genetic association with SSc (2). The MHC classes I and II have been identified as susceptible factors in SSc pathogenesis, and an evident ethnic variation is seen in these associations (2). Studies have reported different or the same HLA alleles as a risk factor or a protective factor in different ethnic populations (2). HLA polymorphism is also linked to susceptibility to a wide range of AIDs such as systemic lupus erythematosus (SLE), rheumatoid arthritis (RA), autoimmune hepatitis (AIH), and so forth (4). Studies have reported HLA alleles as common risk factors in AIDs (4). From India to date, there is no report on the genetics of SSc. HLA associations can be unique for specific populations or common among different ethnic populations. Hence, in the present study, we wish to determine the HLA association with SSc patients having familial and non-familial inheritance and the genetic difference between lcSSc and dcSSc. This will be the first genetic report from India to decipher the role of HLA in SSc pathogenesis.

## 2 Materials and methods

### 2.1 Study population

This is a cross‐sectional study carried out in the Department of Immunopathology, and the SSc patients were recruited from the Department of Internal Medicine, Postgraduate Institute of Medical Research and Education, Chandigarh. One hundred fifty (female 138 and male 12) adult-onset (≥ 18 years) SSc patients, having a mean ± SD age of 40.3 ± 10.9 years, presenting with clinical signs and symptoms without familial history and fulfilling the ACR/EULAR criteria guidelines were enrolled from North India. They were categorized into two clinical subgroups according to the criteria of Le Roy et al. (5) as having lcSSc (*n* = 70) or dcSSc (*n* = 80). The controls consisted of 150 healthy unrelated individuals (female 138 and male 12), having a mean ± SD age of 41.5 ± 9.6 years with North Indian origin similar to the patients. Five North Indian families consisting of parent–child/Sibling pairs affected with SSc or overlap syndrome were also enrolled. This study was approved by the Institutional Ethics Committee (NK/3163/Ph.D./362), and written informed consent was obtained from each study subject.

### 2.2 ANA and autoantibodies tests

The sera from familial SSc patients were tested for antinuclear antibodies (ANA) by indirect immunofluorescence followed by line immunoblot assay for examining the specific autoantibodies, using the same reagents and protocol in our previously published article (6), which describes the ANA pattern and autoantibody profiling of North Indian SSc patients.

### 2.3 Genomic DNA extraction

Genomic DNA was extracted from whole blood using a commercially available kit (QIAamp DNA, Qiagen, Hilden, Germany). A260/A280 measurements assessed the concentration and purity of DNA on a spectrophotometer (Genova Nano, Jenway, UK).

### 2.4 HLA classes I (A and B) and II (DRB1 and DQB1) genotyping in non-familial SSc

The HLA-A, B, DRB1, DQB1 genotyping of 150 patients and 150 controls were performed with LIFE CODES SSO typing kits (Immucor, Norcross, GA, USA) using the Luminex platform (LX200, Austin, TX, USA). The HLA MatchIT DNA software was used in sequencing analysis and assigning HLA alleles.

### 2.5 HLA class I (A, B, and C) and II (DRB1, DQB1, and DPB1) genotyping in familial SSc

HLA typing for HLA classes I (A, B, and C) and II (DRB1, DQB1, and DPB1) for the familial study of SSc was performed by Next Generation Sequencing (NGS) with Illumina NovaSeq6000. HLA genotype was determined based on IMGT/HLA database release 3.42.0.

### 2.6 Statistical analysis

Phenotype frequencies were compared between patients and controls using the chi-square test or Fisher exact test. Bonferroni correction was applied to the *p*-values. Two-sided *P*-values less than 0.05 were considered significant. Analyses were performed using SPSS software (version 20.0).

## 3 Results

HLA types I and II association in patients with familial and non-familial SSc (lcSSc and dcSSc) from North India

### 3.1 HLA-A, HLA-B, HLA-DRB1, and HLA-DQB1 genotyping in non-familial SSc patients

The results of genotyping for HLA-A, HLA-B, HLA-DRB1, and HLA-DQB1 in SSc patients (*n* = 150; 70 lcSSc and 80 dcSSc) and controls (*n* = 150) are summarized in **Tables 1**–**8**.

**Table 1:** Comparison of phenotype frequencies of HLA-A in SSc patients and control.

HLA-A	Patients (*n* = 150) (%PF)	Controls (*n* = 150) (%PF)	*P*-value	*Pc*-value	OR	95% CI
A*01	30 (20%)	35 (23.3%)	0.48	–	0.82	0.474–1.425
A*02	30 (20%)	38 (25.3%)	0.26	–	0.73	0.422–1.257
A*03	20 (14.29%)	20 (14.29%)	–	–	1.00	0.518–1.929
A*11	68 (45.3%)	58 (38.7%)	0.24	–	1.31	0.8259–2.075
A*24	60 (40.0%)	37 (24.6%)	0.004*	0.05	2.03	1.238–3.335
A*26	26 (17.3%)	19 (12.6%)	0.25	–	1.44	0.7691–2.690
A*29	1 (0.7%)	3 (2.0%)	0.31	–	0.32	0.034–3.198
A*30	3 (2.0%)	9 (6.0%)	0.13	–	0.31	0.091–1.196
A*31	5 (3.3%)	11 (7.3%)	0.19	–	0.43	0.164–1.311
A*32	14 (9.3%)	5 (3.3%)	0.03*	0.39	2.98	1.120–7.672
A*33	22 (14.6%)	32 (21.3%)	0.13	–	0.63	0.351–1.145
A*68	15 (10.0%)	30 (20.0%)	0.01*	0.13	0.44	0.228–0.854
A*69	2 (1.3%)	1 (0.7%)	0.33	–	2.01	0.181–22.445

*Statistically significant using Fisher’s exact/chi-square test.

n, number of individuals; %PF, percentage phenotype frequency; OR, odds ratio; 95% CI, 95% confidence interval; Pc, P-value corrected with Bonferroni correction.

**Table 2:** Comparison of phenotype frequencies of HLA-B in SSc patients and control.

HLA-B	Patients (*n* = 150) (%PF)	Controls (*n* = 150) (%PF)	*P*-value	*P*_c_-value	OR	95% CI
B*07	15 (10.0%)	21 (14.0%)	0.28	–	0.68	0.339–1.375
B*08	25 (16.7%)	15 (10.0%)	0.08	–	1.80	0.890–3.510
B*13	12 (8.0%)	13 (8.7%)	0.83	–	0.91	0.394–2.068
B*15	31 (20.6%)	36 (24.0%)	0.48	–	0.82	0.474–1.415
B*18	12 (8.0%)	12 (8.0%)	–		1.00	0.421–2.374
B*27	4 (2.6%)	9 (6.0%)	0.25	–	0.42	0.143–1.392
B*35	55 (36.75%)	33 (22.0%)	0.005*	0.11	2.05	1.252–3.352
B*37	5 (3.3%)	6 (4.0%)	–	–	0.82	0.281–2.878
B*38	5 (3.3%)	3 (2.0%)	0.72	–	1.69	0.438–6.468
B*39	1 (0.7%)	0%	–	–	–	–
B*40	28 (18.6%)	40 (26.7%)	0.09	–	0.63	0.372–1.079
B*41	2 (1.3%)	6 (4.0%)	0.15		0.32	0.065–1.350
B*44	25 (16.6%)	24 (16.0%)	0.87		1.05	0.580–1.911
B*47	1 (0.6%)	1 (0.6%)	–	–	1.00	0.052–19.11
B*50	2 (1.3%)	2 (1.3%)	–	–	1.00	0.155–6.451
B*51	32 (21.3%)	27 (18.0%)	0.46	–	1.23	0.712–2.181
B*52	12 (8.0%)	13 (8.6%)	0.83	–	0.91	0.394–2.068
B*53	0.0%	1 (0.6%)	–	–	0.00	0.000–9.000
B*55	11 (7.3%)	6 (4.0%)	0.21	–	1.89	0.681–5.197
B*56	1 (0.6%)	0.0%	–	–	–	–
B*57	8 (5.3%)	12 (8.0%)	0.34	–	0.64	0.263–1.535
B*58	10 (6.7%)	13 (8.7%)	0.51	–	0.75	0.322–1.792

*Statistically significant using Fisher’s exact/chi-square test.

n, number of individuals; %PF, percentage phenotype frequency; OR, odds ratio; 95% CI, 95% confidence interval; Pc, P-value corrected with Bonferroni correction.

**Table 3:** Comparison of phenotype frequencies of HLA-DRB1 in SSc patients and control.

HLA-DRB1	Patients (*n* = 150) (%PF)	Controls (*n* = 150) (%PF)	*P*-value	*P*_c_-value	OR	95% CI
DRB1*01	10 (6.7%)	10 (6.7%)	–	–	1.00	0.399–2.502
DRB1*03	33 (22.0%)	24 (16.0%)	0.18	–	1.48	0.840–2.661
DRB1*04	17 (11.3%)	24 (16.0%)	0.23	–	0.67	0.337–1.329
DRB1*07	41 (27.3%)	35 (23.3%)	0.42	–	1.23	0.727–2.048
DRB1*08	3 (2.0%)	5 (4.0%)	0.72	–	0.59	0.154–2.283
DRB1*09	2 (1.3%)	3 (2.0%)	–	–	0.66	0.116–3.286
DRB1*10	5 (3.3%)	15 (10.0%)	0.03*	0.39	0.31	0.121–0.819
DRB1*11	50 (33.3%)	26 (17.3%)	0.001*	**0.01**	2.38	1.375–4.037
DRB1*12	0.0%	19 (12.6%)	0.0001*	**0.001**	0.00	0.000–0.166
DRB1*13	32 (21.3%)	26 (17.3%)	0.38	–	1.29	0.740–2.312
DRB1*14	20 (13.3%)	24 (16.0%)	0.51	–	0.80	0.437–1.539
DRB1*15	87 (58.0%)	84 (56.0%)	0.72	–	1.08	0.683–1.727
DRB1*16	0.0%	2 (1.3%)	0.49	–	0.00	0.000–2.158

*Statistically significant using Fisher’s exact/chi-square test; red, risk allele; green, protective allele.

n, number of individuals; %PF, percentage allelic frequency; OR, odds ratio; 95% CI, 95% confidence interval; Pc, P-value corrected with Bonferroni correction.

Bold values indicate statistical significance of p < 0.05 after Bonferroni correction.

**Table 4:** Comparison of phenotype frequencies of HLA-DQB1 in SSc patients and control.

HLA-DQB1	Patients (*n* = 150) (% PF)	Controls (*n* = 150) (%PF)	*P*-value	*P*_c_-value	OR	95% CI
DQB1*02	62 (41.3%)	55 (36.7%)	0.40	–	1.21	0.755–1.914
DQB1*03	79 (52.7%)	82 (54.7%)	0.72	–	0.92	0.583–1.456
DQB1*04	2 (1.3%)	6 (4.0%)	0.28	–	0.32	0.065–1.350
DQB1*05	76 (50.7%)	69 (46.0%)	0.41	–	1.20	0.765–1.906
DQB1*06	80 (53.3%)	88 (58.7%)	0.35	–	0.80	0.505–1.275

n, number of individuals; %PF, percentage phenotype frequency; OR, odds ratio; 95% CI, 95% confidence interval Pc; P-value corrected with Bonferroni correction.

**Table 5:** Comparison of phenotype frequencies of HLA-A in lcSSc and dcSSc patients.

HLA-A	lcSSc (*n* = 70) (% PF)	dcSSc (*n* = 80) (% PF)	*P*-value	*P*_c_-value	OR	95% CI
A*01	15 (21.4%)	15 (18.8%)	0.48	–	0.82	0.474–1.425
A*02	12 (17.1%)	18 (22.5%)	0.41	–	0.71	0.316–1.608
A*03	9 (12.9%)	11 (13.8%)	0.87	–	0.92	0.359–2.383
A*11	34 (48.6%)	34 (42.5%)	0.45	–	1.27	0.670–2.436
A*24	27 (38.6%)	33 (41.3%)	0.73		0.89	0.464–1.723
A*26	13 (18.6%)	13 (16.3%)	0.70	–	1.17	0.504–2.739
A*29	0.0%	1 (1.3%)	0.34	–	1.88	1.622–2.194
A*30	2 (2.9%)	1 (1.3%)	0.48	–	2.32	0.206–26.189
A*31	1 (1.43%)	4 (5.0%)	0.37	–	0.27	0.022–1.739
A*32	7 (10%)	7 (8.7%)	0.9		1.1	0.4238–3.164
A*33	10 (14.2%)	12 (15%)	0.92	–	0.9	0.3708–2.248
A*68	7 (10.0%)	8 (10.0%)	1.00		1.00	0.343–2.913
A*69	1 (1.4%)	1 (1.3%)	0.92	–	1.14	0.070–18.65

n, number of individuals; %PF, percentage phenotype frequency; OR, odds ratio; 95% CI, 95% confidence interval; P_c_, P-value corrected with Bonferroni correction.

**Table 6:** Comparison of phenotype frequencies of HLA-B in lcSSc and dcSSc patients.

HLA-B	lcSSc (*n* = 70) (%PF)	dcSSc (*n* = 80) (%PF)	*P*-value	*P*_c_-value	OR	95% CI
B*07	8 (11.4%)	7 (8.8%)	0.58	–	1.34	0.446–3.577
B*08	10 (14.3%)	15 (18.8%)	0.46	–	0.72	0.298–1.729
B*13	6 (8.6%)	6 (7.5%)	0.80	–	1.15	0.339–3.933
B*15	13 (18.6%)	18 (22.5%)	0.55	–	0.78	0.352–1.783
B*18	7 (10%)	5 (6.3%)	0.39	–	1.66	0.542–4.859
B*27	3 (4.3%)	1 (1.3%)	0.25	–	3.53	0.513–46.45
B*35	24 (34.2%)	31 (38.7%)	0.5		0.8	0.429–1.647
B*37	1 (1.4%)	4 (5.0%)	0.22	–	0.27	0.022–1.739
B*38	4 (5.7%)	1 (1.3%)	0.12	–	4.78	0.756–59.22
B*39	0.0%	1 (1.3%)	0.34	–	0.00	0.000–10.29
B*40	14 (20%)	14 (17.5%)	0.69	–	1.17	0.521–2.664
B*41	0.0%	2 (2.5%)	0.18	–	0.00	0.000–2.463
B*44	10 (14.3%)	15 (18.7%)	0.5		0.7	0.298–1.729
B*47	0.0%	1 (1.3%)	0.34	–	0.00	0.000–10.29
B*50	1 (1.4%)	1 (1.3%)	0.92	–	1.14	0.0594–22.00
B*51	17 (24.3%)	15 (18.8%)	0.4	–	1.39	0.619–2.923
B*52	3 (4.2%)	9 (11.2%)	0.1	–	0.3	0.100–1.392
B*53	0.0%	0.0%	–	–	–	–
B*55	5 (7.1%)	6 (7.5%)	0.93	–	0.948	0.310–3.448
B*56	0.0%	1 (1.3%)	–	–	0.00	0.000–10.29
B*57	6 (8.57%)	2 (2.5%)	0.14	–	3.65	0.873–18.16
B*58	7 (10%)	3 (3.8%)	0.12	–	2.85	0.740–10.41

n, number of individuals; %PF, percentage phenotype frequency; OR, odds ratio; 95% CI, 95% confidence interval; P_c_, P-value corrected with Bonferroni correction.

**Table 7:** Comparison of phenotype frequencies of HLA-DRB1 in lcSSc and dcSSc patients.

HLA-DRB1	lcSSc (*n* = 70) (%PF)	dcSSc (*n* = 80) (%PF)	*P*-value	*P*_c_-value	OR	95% CI
DRB1*01	5 (7.1%)	5 (6.2%)	–	–	1.15	0.355–3.742
DRB1*03	15 (21.4%)	18 (22.5%)	0.87	–	0.93	0.451–2.05
DRB1*04	5 (7.1%)	12 (15%)	0.12	–	0.43	0.163–1.329
DRB1*07	22 (31.4%)	19 (23.7%)	0.29	–	1.47	0.698–2.946
DRB1*08	1 (1.4%)	2 (2.5%)	–	–	0.56	0.038–4.961
DRB1*09	2 (2.8%)	0.0%	0.21	–	–	–
DRB1*10	2 (2.8%)	3 (4.2%)	–	–	0.65	0.114–3.310
DRB1*11	25 (35.7%)	25 (31.2%)	0.56	–	1.22	0.627–2.386
DRB1*12	0.0%	0.0%	–	–	–	–
DRB1*13	14 (20%)	18 (22.5%)	0.70	–	0.86	0.4015–1.918
DRB1*14	9 (12.8%)	11 (13.7%)	0.87	–	0.92	0.385–2.387
DRB1*15	41 (58.5%)	46 (57.5%)	0.89	–	1.04	0.540–2.038
DRB1*16	0.0%	0.0%	–	–	–	–

n, number of individuals; %PF, percentage phenotype frequency; OR, odds ratio; 95% CI, 95% confidence interval; P_c_, P-value corrected with Bonferroni correction.

**Table 8:** Comparison of phenotype frequencies of HLA-DQB1 in lcSSc and dcSSc patients.

HLA-DQB1	lcSSc (*n* = 70) (%PF)	dcSSc (*n* = 80) (%PF)	*P-*value	*P*_c_-value	OR	95% CI
DQB1*02	30 (42.8%)	32 (40%)	0.72	–	1.12	0.57–2.19
DQB1*03	38 (54.2%)	41 (51.2%)	0.71	–	1.13	0.592–2.168
DQB1*04	1 (1.4%)	1 (1.2%)	0.92	–	1.14	0.059–22.00
DQB1*05	32 (45.7%)	44 (55%)	0.25	–	0.68	0.356–1.311
DQB1*06	37 (52.8%)	43 (53.7%)	0.91	–	0.96	0.504–1.846

n, number of individuals; %PF, percentage phenotype frequency; OR, odds ratio; 95% CI, 95% confidence interval; P_c_, P-value corrected with Bonferroni correction.

Phenotype (*P* = 0.001, OR: 2.38, CI: 1.375–4.037, *P*_c_ = 0.01) frequency of HLA-DRB1*11 was significantly high in SSc patients compared with controls suggesting their role in disease susceptibility.

The phenotype frequencies of HLA-A*24 (*P* = 0.004, OR: 2.03, CI: 1.238–3.335, *P*_c_ = 0.05), HLA-A*32 (*P* = 0.03, OR: 2.98, CI: 1.120–7.672, *P*_c_ = 0.39), and HLA-B*35 (*P* = 0.005, OR: 2.05, CI: 1.252–3.352, *P*_c_ = 0.11), were observed to be high in SSc patients. However, after applying the Bonferroni correction, the *P*-value lost its significance.

Phenotype (*P* = .0001, OR: 0.00, CI: 0.000–0.166, *P*_c_ = 0.001) frequency of HLA-DRB1*12 was significantly less prevalent in SSc patients compared with controls suggesting a protective association.

The phenotype frequencies of HLA-A*68 (*P* = 0.01, OR: 0.44, CI: 0.228–0.854, *P*_c_ = 0.13) and HLA- DRB1*10 (*P* = 0.03, OR: 0.31, CI: 0.121–0.819, *P*_c_ = 0.39), were found to be less prevalent in SSc patients than controls; however, the association did not attain statistical significance after the Bonferroni correction.

None of the HLA-DQB1 alleles were found to be associated with SSc patients compared with controls.

### 3.2 HLA-A, HLA-B, HLA-DRB1, and HLA-DQB1 phenotype frequency in lcSSc and dcSSc patients

Stratification of genotyping results into the limited and diffused form of SSc revealed no significant difference in the allele frequencies among the two groups (**Tables 5**–**8**).

### 3.3 HLA classes I (A, B, and C) and II (DRB1, DQB1, and DPB1) genotyping in familial SSc

**Figure 1:**
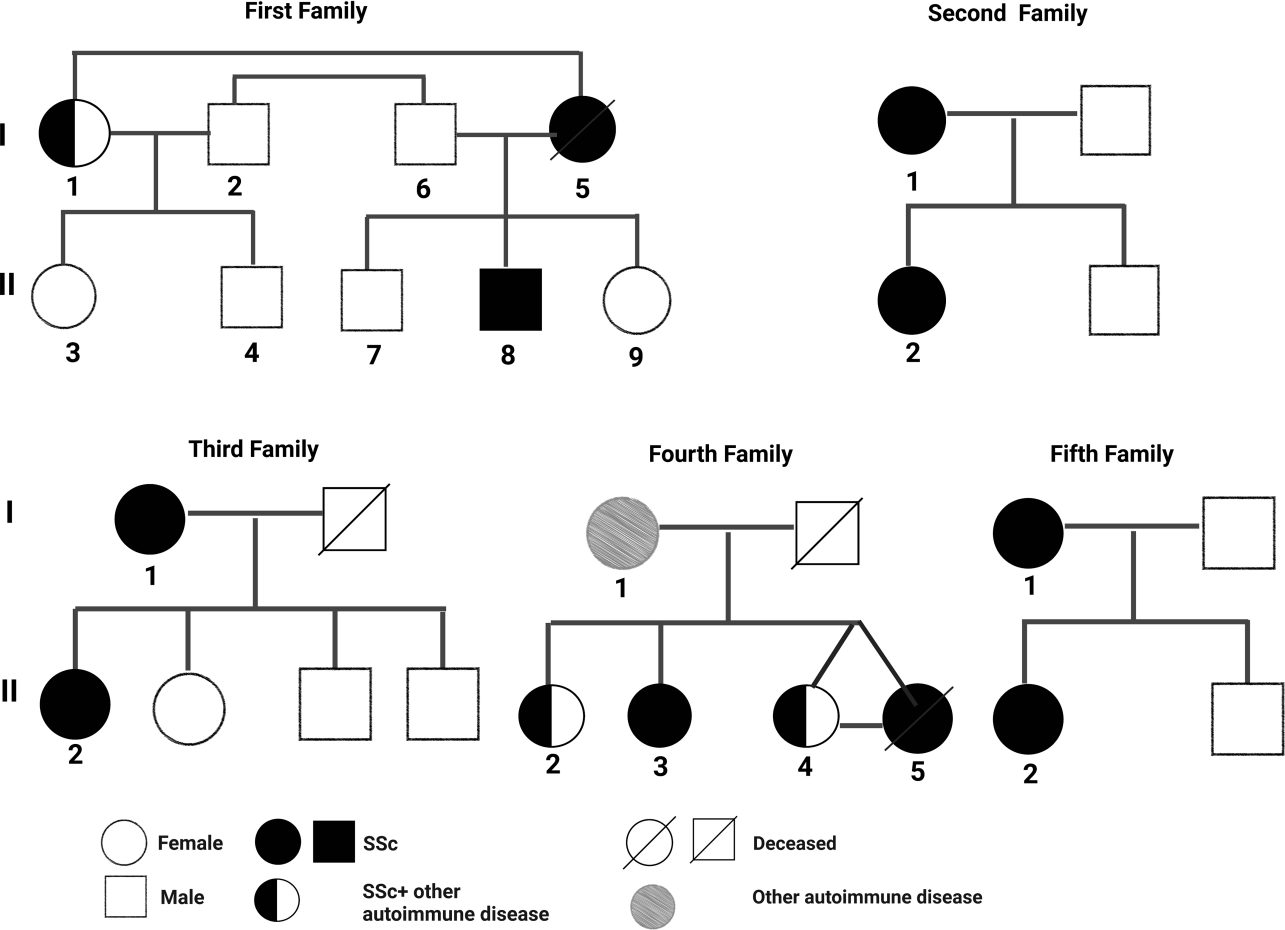
Pedigree of five families having familial SSc patients.

The pedigree, demographic-clinical details and HLA haplotype of five families are shown in **Figure 1** and **Tables 9** **,** **10**.

**Table 9:** Demographic-clinical details of 1st, 2nd, 3rd, 4th, and 5th family.

1st Family (Himachal Pradesh, India)				
Subject	Age (years)	Clinical diagnosis/ age at diagnosis (years)	ANA pattern	Associated autoAbs
**I-1**	42 years	SLE (34 years), lcSSc (42 years)	Speckled	Negative
**I-2**	48 years	Healthy	Negative	Negative
**II-3**	20 years	Sensitivity to cold water (19 years)	Negative	Negative
**II-4**	24 years	Healthy	Negative	Negative
**I-5**	44 years (deceased)	dcSSc (36 years)	*NA	*NA
**I-6**	51 years	Healthy	Negative	Negative
**II-7**	23 years	Healthy	Negative	Negative
**II-8**	28 years	dcSSc with Raynaud’s phenomenon, skin tightening, and ILD (20 years)	Topo I like pattern	Topo I
**II-9**	30 years	Healthy	Negative	Negative
2nd Family (Punjab, India)				
**I-1**	40 years	dcSSc with Raynaud’s phenomenon, ILD, and dyspnea (29 years)	Topo-I like pattern	Topo I, nucleolus organizer region (NOR)-90, Pm-Scl75
**II-2**	23 years	dcSSc with Raynaud’s phenomenon, skin tightening, ILD, and digital ulcer (18 years)	Topo-I like pattern	Topo I
3rd Family (Chandigarh, India)				
**I-1**	53 years	lcSSc with PAH (50 years)	Centromeric pattern	CENP-A, CENP-B, Ro-52, and RP11
**II-2**	34 years	dcSSc with ILD and PAH (26 years)	Topo I like pattern	Topo I, and Ro-52
4th Family (Chandigarh, India)				
**I-1**	83 years	RA (40 years)	Negative	Negative
**II-2**	58 years	SLE (42 years), myositis, dcSSc (57 years)	Speckled pattern	Ro-52
**II-3**	54 years	SSc	*NA	*NA
**II-4**	50 years	RA (38 years), SSc, breast cancer	Speckled pattern	Ro-52, Pm-Scl75
**II-5**	44 years (deceased)	dcSSc with ILD (30 years**)**	*NA	*NA
5th Family (Chandigarh, India)				
**I-1**	52 years	Progressive SSc with skin tightening, Raynaud’s phenomenon, ILD, and dyspnea (40 years)	Speckled pattern	CENP A, RO-52, ku
**II-2**	24 years	SSc with Raynaud’s phenomenon and skin tightening (24 years)	Speckled pattern	Ku

ANA, antinuclear antibodies; autoAbs, autoantibody; SLE, systemic lupus erythematosus; dcSSc, diffuse cutaneous systemic sclerosis; *NA, sample not available; ILD, Interstitial lung disease; Topo I, Topoisomerase I; lcSSc, limited cutaneous systemic sclerosis; PAH, pulmonary arterial hypertension; CENP-A, Centromere A; CENP-B, Centromere B; RA, rheumatoid arthritis.

**Table 10:** HLA haplotypes of 1st, 2nd, 3rd, 4th, and 5th family.

MHC	Class I			Class II		
Subjects	A	B	C	DRB1	DQB1	DPB1
1st Family						
**I-1**	11:01:01 32:01:01	51:01:01 55:01:01	14:02:01 04:01:01	15:06:01 04:08:01	05:02:01 03:02:01	04:01:01 04:01:01
**I-2**	11:01:01 24:02:01	35:01:01 15:01:01	04:01:01 04:01:01	15:06:01 15:06:01	05:02:01 05:02:01	02:01:02 26:01:02
**II-3**	11:01:01 24:02:01	35:01:01 55:0101	04:01:01 04:01:01	15:06:01 15:06:01	05:02:01 05:02:01	04:01:01 26:01:02
**II-4**	32:01:01 11:01:01	51:01:01 15:01:01	14:02:01 04:01:01	15:06:01 04:08:01	05:02:01 03:02:01	02:01:02 04:01:01
I-5*	11:01:01 32:01:01	51:01:01 55:01:01	14:02:01 04:01:01	15:06:01 04:08:01	05:02:01 03:02:01	04:01:01 04:01:01
**I-6**	24:02:01 33:03:01	35:01:01 44:03:02	04:01:01 07:06	15:06:01 07:01:01	05:02:01 02:02:01	26:01:02 14:01:01
**II-7**	11:01:01 24:02:01	35:01:01 55:01:01	04:01:01 04:01:01	15:06:01 15:06:01	05:02:01 05:02:01	04:01:01 26:01:02
**II-8**	11:01:01 24:02:01	35:01:01 55:01:01	04:01:01 04:01:01	15:06:01 15:06:01	05:02:01 05:02:01	04:01:01 26:01:02
**II-9**	24:02:01 32:01:01	51:01:01 35:01:01	14:02:01 04:01:01	04:08:01 15:06:01	03:02:01 05:02:01	26:01:02 04:01:01
2nd Family						
**I-1**	11:01:01 33:03:01	44:03:02 52:01:01	07:06:01 12:02:02	**11**:04:01 07:01:01	02:02:01 03:01:01	04:01:01 13:01:01
**II-2**	11:01:01 33:03:01	44:03:02 52:01:01	07:06:01 12:02:02	**11**:04:01 07:01:01	03:03:02 03:01:01	04:01:01 13:01:01
3rd Family						
**I-1**	11:01:01 11:01:01	35:01:01 35:01:01	04:01:01 04:01:01	15:06:01 15:06:01	05:02:01 05:02:01	03:01:01 03:01:01
**II-2**	11:01:01 11:01:01	35:01:01 35:01:01	04:01:01 04:01:01	15:06:01 15:06:01	05:02:01 05:02:01	03:01:01 13:01:01
4th Family						
**I-1**	03:01:01 24:02:01	08:01:01 40:06:01	07:02:01 15:02:01	03:01:01 **11**:01:01	02:01:01 03:01:01	04:01:01 26:01:02
**II-2**	03:01:01 68:01:02	08:01:01 40:06:01	07:02:01 15:02:01	03:01:01 04:01:01	02:01:01 03:02:01	03:01:01 26:01:02
**II-4**	03:01:01 68:01:02	08:01:01 40:06:01	07:02:01 15:02:01	03:01:01 04:01:01	02:01:01 03:02:01	03:01:01 26:01:02
II-5*	03:01:01 68:01:02	08:01:01 40:06:01	07:02:01 15:02:01	03:01:01 04:01:01	02:01:01 03:02:01	03:01:01 26:01:02
5th Family						
**I-1**	02:05:01 11:01:01	18:01:01 50:01:01	06:02:01 07:01:01	15:01:01 15:01:01	06:01:01 06:02:01	04:01:01 04:01:01
**II-2**	02:05:01 03:01:01	50:01:01 51:01:01	06:02:01 14:02:01	13:01:01 15:01:01	06:02:01 06:02:01	03:01:01 04:01:01

(White row, diseased; gray row, healthy; orange row, symptomatic without clinical evidence; maroon, allele also associated with non-familial SSc patients).

Note: *The haplotype of subject I-5 (1st Family) was predicted through her spouse’s (subject I-6) and children’s (subject I-7/8/9) haplotype; The haplotype of subject II-5 (4th Family) was predicted through her twin sister’s (subject II-4) haplotype (monozygotic twin).

The risk allele HLA-DRB1*11 associated with the non-familial SSc patients was present in the SSc-affected family members of the second family. The protective allele HLA-DRB1*12 associated with the non-familial SSc patients was absent in all the SSc-affected family members.

## 4 Discussion

This is the first report on the HLA association with SSc from India. We investigated HLA types I and II association in patients with familial and non-familial SSc from North India. We also examined the HLA association within the subgroup of SSc (lcSSc *vs.* dcSSc). In this study, the HLA-DRB1*11 allele was significantly predisposing in SSc patients compared with the controls, while the HLA-DRB1*12 allele emerged as a protective allele. SSc’s most consistent genetic risk factor reported is the HLA-DRB1*11 alleles found in different ethnic populations. HLA-DRB1*11 has a strong genetic association among Caucasians, Spanish, and Greece populations (7–10). Also, in a large multicentric study involving three ethnic groups (Caucasian, Black, and Hispanic) in the United States, which covered 1,300 patients, the strongest association in the Caucasian and Hispanic subjects was with HLA-DRB1*11:04 haplotype; they also found DRB1*11:04 allele association with anti–topo-I positivity (11). In the present study, HLA-DRB1*11 showed the strongest association with SSc, further confirming HLA-DRB1*11 as a susceptibility allele for SSc. There are no previous reports on the association of the protective allele HLA-DRB1*12 with SSc in other ethnic populations. However, HLA-DRB1*12 is reported to be a protective allele against type 1 Diabetes (T1D) and Graves’ disease in the Taiwan population (12). Possibly genetic heterogeneity among ethnicities might be significantly impacting the complex trait of SSc. Previous studies of different populations already implicated ethnic differences in genetic association with SSc (2, 13), while some share the same genetic determinant as in the case of HLA-DRB*11 (7–10). Therefore, HLA-DRB1*12 is unique to our North Indian population of SSc. None of the HLA-DQB1 alleles were associated with North Indian SSc patients, unlike other ethnic populations (11, 14, 15). We also did not find any significant difference in the phenotype frequency among the two subsets of SSc (lcSSc *vs.* dcSSc), again, which could be due to ethnic variations. **Table 11** shows the HLA association with SSc in the different ethnic populations.

**Table 11:** HLA genes associated with SSc.

HLA-associated genes	Population (number)	Reference
Risk allele		
A*30	Brazilian (141)	(16)
B*13	Caucasian (95)	(7)
B*35	Brazilian (141) Choctaw Indian (12)	(16) (17)
B*62	Caucasian (95)	(7)
B*65	Caucasian (95)	(7)
C*04	Brazilian (141)	(16)
Cw4	Choctaw Indians (12)	(17)
Cw*0602	Caucasian (95)	(7)
DPB1*03:01	Chinese (338) Japanese (463)	(13) (18)
DPB1*1301	Chinese (338) Korean (1,107)	(13) (19)
DPB1*0901	Korean (1,107) Japanese (463)	(19) (18)
DQA1 *0501	Caucasian (86) Italian (392) Multi-ethnic US cohort (1300; 961-White, 178-Black, and 161-Hispanic)	(20) (14) (11)
DQB1*03:01	Italian (392) Spanish (922) Multi-ethnic US cohort (1300; 961-White, 178-Black, and 161-Hispanic)	(14) (14) (11)
DQB1*03:03	Chinese (213)	(15)
DQBl*05:01	Chinese (213) Japanese (463)	(15) (18)
DQBl*06:11	Chinese (213)	(15)
DRB1*01	Caucasian (95)	(7)
DRB1*08:04	African American (662)	(21)
DRB1*11	Caucasian (95)	(7)
DRB1*1104	African American (662) Italian (392) Spanish (922) Multi-ethnic US cohort (1300; 961-White, 178-Black, and 161-Hispanic)	(21) (14) (14) (11)
DRB1*1502	Thai (50) Japanese (463)	(22) (18)
DRB1*1602	Choctaw Indians (12)	(17)
DRB1*0407	Caucasian (1,083), African American (177), and Hispanic (208)	(23)
DRB1*1304	Caucasian (1,083), African American (177), and Hispanic (208)	(23)
DRB5*01:02	Thai (50)	(22)
Protective allele		
B*57	Caucasian (95)	(7)
C*03	Brazilian (141)	(16)
HLA-Cw* 14	Caucasian (95)	(7)
DPB1*02:01	Japanese (463)	(18)
DQA1*0201	Multi-ethnic US cohort (1300; 961-White, 178-Black, and 161-Hispanic)	(11)
DQB1*0202	Multi-ethnic US cohort (1300; 961-White, 178-Black, and 161-Hispanic)	(11)
DQB1∗03:01	Japanese (463)	(18)
DQB1*05	Brazilian (141)	(16)
DRB1*01	Brazilian (141)	(16)
DRB1*07	Caucasian (95)	(7)
DRB1*0701	Multi-ethnic US cohort (1300; 961-White, 178-Black, and 161-Hispanic)	(11)
DRB1∗13:02	Japanese (463)	(18)
DRB1∗14:06	Japanese (463)	(18)
DRB1*1501	Multi-ethnic US cohort (1300; 961-White, 178-Black, and 161-Hispanic)	(11)

The risk allele HLA-DRB1*11, found to be associated with the non-familial SSc cohort, was present only in the SSc-affected family members of the second family. The mother–daughter not only shared a risky HLA haplotype (HLA-DRB1*01) for SSc but also manifested a similar disease subset (dcSSc), and both were positive for SSc-specific topo-1 antibody. This is in line with prior studies indicating that the type of HLA can define the disease subset and autoantibodies (21, 24). The protective allele HLA-DRB1*12 found in non-familial SSc patients was absent in all the familial SSc patients investigated.

Interestingly, in the 3rd family, the daughter (subject II-1) had clinical signs and symptoms of dcSSc earlier at 26 years, and the mother (subject I-1) was diagnosed with lcSSc later at the age of 50 years. Also, both had a different set of multiple autoAbs. The possible reason could be the presence of the HLA-DPB1*13 allele in the daughter, making her more susceptible towards the SSc; the rest of the HLA haplotypes were the same in both mother and daughter. HLA-DPB1*13 allele is a known risk factor for SSc (13, 19) and is also associated with the topo-I autoantibody subset of SSc in the European American ancestral cohort (21). In our North Indian cohort of SSc, we found topo-I autoAb to be associated with the dcSSc and clinical complications such as Raynaud’s, ILD, and PAH (6). The daughter had a serological presence of topo-I autoAb. Altogether, the HLA-DPB1*13 allele in the daughter could be the possible reason for the existence of topo-I autoAb, causing early disease onset and development of a more severe subset of SSc compared with the mother.

Our study shows three cases in familial SSc patients where SSc was co-existing with other AIDs. Subject I-1 (1st family) had both SLE and lcSSc, subject II-2 (4th family) had SLE, myositis, APLA, and dcSSc, a case of polyautoimmunity, and subject II-4 (4th family) had RA and SSc. A meta-analysis study by Cruz-Tapias et al. identified HLA-DRB1*03 and HLA-DRB1*04 as common alleles conferring susceptibility to more than one AIDs: RA, SLE, AIH, MS (multiple sclerosis), and T1D (25). The presence of DRB1*04 in subject I-1 (1st family) and DRB1*03 and DRB1*04 in subject II-2,4 (4th family) might be the reason for the occurrence of distinct AIDs within an individual (polyautoimmunity) and within members of a nuclear family (familial autoimmunity), supporting the concept of a shared autoimmune genetic background among AIDs.

The findings partially explain the clustering of the disease within families, suggesting the role of non-HLA genes, environmental factors, and epigenetic influences in disease development.

Due to the resource-limited setting, we could not perform HLA-C and HLA-DPB1 genotyping and used a low-resolution detection kit for HLA genotyping in non-familial SSc patients, the major limitation of our study.

## 5 Conclusion

The HLA-DRB1*11 allele was found to be strongly associated with SSc risk in the non-familial cohort, whereas HLA-DRB1*12 was found to be protective; however, it partially explains the disease clustering in familial SSc patients, suggesting the role of many other factors such as non-HLA genes, epigenetics, and environmental factors, which needs to be further investigated. The present study also suggests HLA sharing in distinct AIDs resulting in polyautoimmunity and familial autoimmunity. Being the initial genetic report from India, this research provides a basis for future studies aiming to understand the complex etiopathogenesis of SSc and explore ethnic variations.

## Data availability statement

The datasets presented in this study can be found in online repositories. The names of the repository/repositories and accession number(s) can be found below: Figshare, https://figshare.com/articles/dataset/HLA_class_I_A_B_C_and_class_II_DRB1_DQB1_DPB1_genotyping_data_in_familial_systemic_sclerosis_by_Next-Generation_Sequencing/24005574.

## Ethics statement

The studies involving human participants were reviewed and approved by Postgraduate Institute of Medical Research and Education, Institutional Ethics Committee (NK/3163/Ph.D./362). The patients/participants provided their written informed consent to participate in this study.

## Author contributions

SM: executed the study, was involved in sample collection, processing and experiments, analyzed the data and wrote the manuscript. SKS: provided the clinical samples and assisted in the clinical classification of patients, and reviewed the manuscript. YK: analyzed the data and reviewed the manuscript. SS: provided the clinical samples and relevant clinical information and reviewed the manuscript. RA: analyzed the data and reviewed the manuscript. SA: contributed to the execution of ANA screening and immunoblotting of familial SSc patients. MK: contributed to the execution of HLA typing. HS: contributed to the execution of HLA typing. RM: responsible for study conception, design, drafting of the manuscript, and data analysis. All authors contributed to the article and approved the submitted version.
